# A Secure, Scalable Large Language Model–Based System (CIDER) for High-Throughput Clinical Data Extraction From Medical Reports: Retrospective Validation Study

**DOI:** 10.2196/95780

**Published:** 2026-09-02

**Authors:** Máté Posta, Aida Figler, Zsófia Dobolyi, Balázs Győrffy

**Affiliations:** 1 Institute of Molecular Life Sciences, HUN-REN Research Centre for Natural Sciences Budapest Hungary; 2 Department of Bioinformatics Semmelweis University Budapest Hungary; 3 Institute of Transdisciplinary Discoveries Medical School University of Pécs Pécs Hungary

**Keywords:** large language model, LLM, natural language processing, NLP, oncology, biobanking, histopathology, health care records, patient documentation, free-text documents

## Abstract

**Background:**

A substantial proportion of clinically relevant information remains locked in unstructured narrative documents, creating a bottleneck for clinical research, biobank annotation, registry development, and real-world evidence generation. While large language models (LLMs) enable advanced clinical text mining, adoption is constrained by concerns regarding data security, multilingual performance, and reproducibility. Manual data abstraction remains predominant for registry curation and retrospective research, despite being labor intensive, costly, and prone to variability.

**Objective:**

We developed and validated CIDER (Clinical Data Extractor), a secure, institutionally deployable, LLM-based pipeline for automated structured data extraction from routine clinical reports. We assessed the potential utility of the system for improving the completeness of clinical research datasets.

**Methods:**

CIDER uses an asynchronous FastAPI-based architecture with a locally deployed vLLM inference engine running Qwen3-VL-32B-Instruct-FP8 model in an institution-controlled environment. The system was validated on 2073 real-world Hungarian-language histopathology reports (a challenging non-English setting), using a manually curated structured database as the reference standard. Seven variables were evaluated (sex, surgery year, T stage, N stage, organ, histology, and size). Extraction performance was assessed using exact-match accuracy, weighted *F*_1_-scores, Cohen κ statistics, and tolerance-based agreement thresholds for tumor size. Robustness was evaluated across temperatures from 0 to 2.0, and technical reproducibility was assessed at a temperature of 0.1 across 3 independent runs.

**Results:**

The validation dataset comprised stand-alone native-text PDF pathology reports originating from multiple Hungarian oncology centers. Input document length showed a median of 3926 (mean 4258, SD 1057, IQR 3490-4688) tokens, while generated outputs contained a median of 63 (mean 62.5, SD 8.2, IQR 56-66) tokens. At a temperature of 0.1, CIDER achieved near-human agreement with expert-curated reference database, with exact-match accuracies of 99.5% for sex, 98.1% for surgery year, 95.8% for organ, 95.6% for T stage, 92.4% for N stage, 87.5% for histology, and 78.1% for tumor size. Weighted *F*_1_-scores ranged from 0.87 for histology to 0.995 for sex, while Cohen κ values ranged from 0.85 for N stage to 0.99 for sex. For tumor size extraction, 83.5% to 85.3% and 87.3% to 88.7% of predictions were within 5 mm and 10 mm of the manually curated values, respectively. CIDER additionally generated candidate extractions for variables omitted during manual curation, including 62.8% (713/1136) of missing T stages and 91.5% (289/316) of tumor size values. Sensitivity testing revealed high robustness, with negligible variance at temperature=0.1 and stable performance at high temperatures (temperature=2.0).

**Conclusions:**

CIDER demonstrates that locally deployed open-weight LLMs can reliably extract structured clinical data from complex pathology reports while preserving institutional control over sensitive data. These findings support the feasibility of secure, institutionally deployable, LLM-based extraction systems for generating research-ready datasets, facilitating clinical registry development, improving dataset completeness, and enabling scalable reuse of unstructured clinical documentation.

## Introduction

The digitalization of modern health care has resulted in an unprecedented accumulation of clinical data. It is estimated that at least 80% of this information remains trapped in unstructured, narrative documents [1,2]. Clinical reports, discharge summaries, and laboratory and histopathology reports contain detailed longitudinal information that is essential not only for medical follow-up but also for prospective and retrospective research and the development of high-quality clinical registries [3]. However, the inherent variability in medical linguistics—characterized by nonstandard abbreviations, complex syntax, frequent grammatical errors, and domain-specific terminology—makes automated data extraction a painstaking challenge in medical informatics [4,5].

Traditionally, the conversion of narrative texts into structured variables was based on manual medical record review [6,7]. While the labor-intensive medical record review is often considered the gold standard for data accuracy, it is profoundly limited by high costs [7], low throughput, and susceptibility to human fatigue, which can lead to errors in large-scale cohorts [8]. Previous attempts to automate this process using rule-based natural language processing (NLP) or early machine learning models often required extensive manual feature engineering and lacked the flexibility to adapt to different medical fields or languages [9,10].

Early automated extraction of clinical information relied on symbolic, rule-based systems, such as MetaMap [11,12] and the clinical Text Analysis and Knowledge Extraction System (cTAKES) [13], which used predefined medical ontologies and regular expressions to identify clinical entities [14]. While these systems achieved high precision in specific, well-defined domains, they still required labor-intensive customization and struggled with the linguistic variability and context dependence inherent in narrative medical texts [10].

With the advent of the transformer architecture [15], there has been a significant shift toward deep learning approaches [16]. Literature has increasingly demonstrated that models pretrained on large-scale medical cohorts (such as Bidirectional Encoder Representations from Transformers [BERT] for biomedical text mining [BioBERT] [17] or clinical BERT [18]) outperform traditional methods by capturing complex semantic relationships within clinical documentation [19]. Recent studies have successfully applied these models to extract tumor node metastasis stages, histology, and biomarker status from pathology reports in English-centric datasets [20-22]. However, despite the fact that only a minority of cases are from English-speaking countries, research focusing on non-English clinical records remains relatively sparse [23].

The emergence of large language models (LLMs) represents a paradigm shift in clinical NLP. These models, trained on massive corpora, exhibit advanced contextual inference capabilities and a high degree of “zero-shot” or “few-shot” adaptability to specialized domains [24-26]. Despite their potential, the adoption of LLMs in clinical environments remains hindered by 3 critical barriers: data security, multilingual performance, and reproducibility [27,28]. The use of commercial, cloud-based LLM services would necessitate the transfer of sensitive health information to external companies and servers, raising significant ethical and legal concerns regarding patient privacy and General Data Protection Regulation (GDPR) compliance [29,30]. Additionally, while frontier models show high proficiency in English, their performance often drops when processing other languages [31,32], particularly within specialized clinical domains such as oncology.

To bridge this gap, we present CIDER (Clinical Data Extractor), an end-to-end system designed for the secure, high-throughput extraction of structured clinical data. CIDER is designed to transform unstructured clinical reports in real time into standardized datasets ready for statistical analysis. The schema-guided framework was designed to support flexible extraction of heterogeneous variables without task-specific retraining. The aim of the present study was to validate the performance of CIDER for extracting clinically relevant information from Hungarian-language oncological histopathology reports and to assess the feasibility of integrating institutionally deployable open-weight LLMs into clinical data curation workflows for research and registry development.

## Methods

### CIDER System Architecture

The CIDER framework is established as a high-throughput, secure clinical data extraction pipeline, developed primarily in Python using the FastAPI framework. To ensure strict compliance with medical data privacy, ethical standards, and full data sovereignty, the system is designed to operate entirely within an on-premises, air-gapped institutional environment. The backend uses asynchronous request handling to maintain high availability and rapid response times, which is essential for processing comprehensive document batches.

The system is deployed on local institutional servers equipped with NVIDIA A40 graphics processing units (GPUs). The core extraction engine uses the open-weight Qwen3-VL-32B-Instruct-FP8 model [33-35], a 32 billion parameter vision-language model selected because it combines multilingual support, strong instruction-following capabilities, and compatibility with institutionally deployable inference workflows. Model selection was based primarily on deployment feasibility and multilingual applicability rather than formal benchmarking against alternative architectures within this study. Although the A40 architecture does not provide native FP8 acceleration, the reduced-precision representation decreased memory requirements and enabled practical deployment of the 32B-parameter model within the available institutional infrastructure. Recent large-scale studies evaluating reduced-precision LLM inference have further shown that FP8-based representations can preserve model quality with minimal or negligible degradation relative to higher-precision formats while improving computational efficiency [36,37].

Model inference is managed via the vLLM [38] serving framework, which exposes an OpenAI-compatible API. This architecture enables efficient memory use while supporting high-throughput, concurrent processing of clinical documents.

The file processing workflow is fully asynchronous to maximize throughput. Upon upload, PDF documents can be processed individually or grouped based on patient-specific metadata when multi-document analysis is required. Text extraction is performed using the *pymupdf* (fitz) library, which parses the unstructured PDF content into machine-readable text. Each file cluster is treated as an independent processing unit.

**Figure 1:**
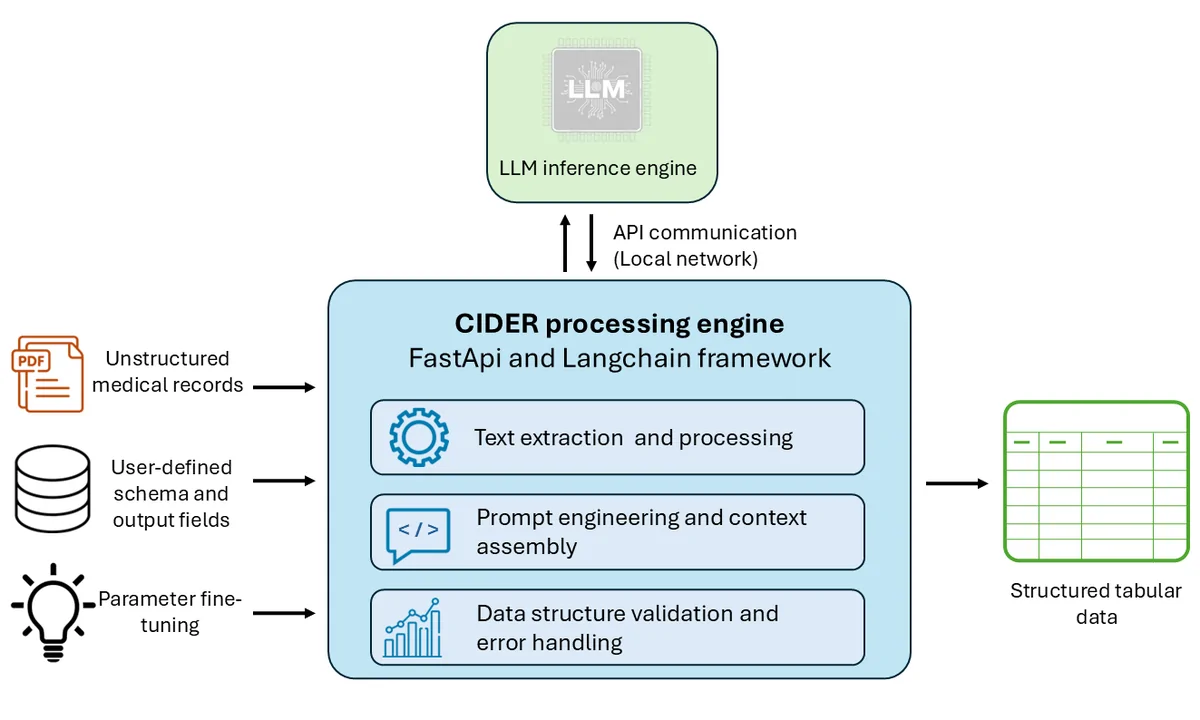
The CIDER (Clinical Data Extractor) system architecture and data extraction workflow. Unstructured medical records in PDF format are processed through a central server. Clinical variables and model parameters (eg, temperature) can be defined, and the system handles data processing, schema-guided extraction, and structured output definition. The final output is a structured, research-ready tabular Excel table.

The final extraction stage integrates prompt assembly with schema-driven generation. Predefined and user-defined data columns are transformed into a rigorous schema, which works as a structural constraint. Starting from a medical record as an input, the model’s output is streamed back to the user interface in a structured JSON format for immediate clinical or research application (Figure 1).

For the inference layer, the *vllm* library is used as the high-performance serving engine. The web application and API management are handled by *fastapi* package. Extraction logic is implemented using the *langchain* framework. For document processing, the *pymupdf* (fitz) library is used.

In the evaluated configuration, all components involved in clinical processing—including document parsing, prompt generation, model inference, and structured output generation—were executed on institution-controlled hardware without transmitting clinical documents or extracted outputs to external cloud services or third-party APIs. The LLM inference engine, FastAPI backend, and supporting processing components were hosted on local GPU servers, and the validation dataset remained within the institutional infrastructure throughout processing. The publicly accessible URL serves as a user-facing interface, while all document processing and model inference were performed on institution-controlled infrastructure. Uploaded documents were processed within temporary storage and removed after extraction. Operational logs contained metadata only (eg, time stamps, session identifiers, filenames, token counts, processing duration, and error information), while report text bodies and extracted clinical values were not stored in operational logging systems. No external cloud-based clinical processing APIs or third-party telemetry services were used during runtime inference.

### Schema-Guided Extraction and Adaptive Prompting

The precision of the CIDER system relies on a multilayered prompt assembly strategy designed to ensure high clinical accuracy and consistency. The extraction pipeline uses a hierarchical prompt structure that combines general extraction rules with project-specific logic and dynamic user constraints.

The backbone of the application is the CIDER’s system prompt (Multimedia Appendix 1), which serves as a robust clinical framework that enforces medical accuracy, consistency, and standard terminology across diverse document types. The system dynamically injects user-defined column descriptions (Multimedia Appendix 2) into the prompt. These descriptions are treated as authoritative rules, allowing the system to adapt its extraction logic based on the specific requirements of a research project without modifying the underlying code.

In this study, both the system prompt and dynamically inserted schema definitions were written in English, whereas the input pathology reports were written in Hungarian. English prompts were selected because contemporary multilingual LLMs are predominantly trained and instruction-tuned on English-centric corpora, and previous work suggests that English prompting may support robust cross-lingual reasoning [39]. Recent evidence suggests that although prompts translated into the target task language may provide marginally better multilingual robustness than English prompts, English prompts remain practically attractive in multilingual environments [40]. As prompt language optimization was not systematically evaluated in the present work, English prompting should be interpreted as an implementation choice rather than an optimized design decision.

### OnkoBank Database Setup Validation

The validation of CIDER was performed using a high-quality, manually curated dataset derived from the institutional OnkoBank database. The OnkoBank cohort included pathology reports originating from several Hungarian oncology treatment centers, thereby introducing variability in reporting practices, terminology, document structure, and writing style. This cohort consists of histopathological reports from 2073 patients who underwent surgical resection for confirmed or clinically suspected malignant neoplasms. Eligible cases included consecutive pathology reports available within the OnkoBank registry between 2022 and 2025. Reports containing native text suitable for automated processing were included in the validation cohort. All medical records related to tissue specimens were collected in connection with surgical procedures performed between 2022 and 2025.

The database serves as a rigorous gold standard due to its 2-stage expert validation process. Initially, the histopathological evaluation and the subsequent medical reports were generated by board-certified pathologists specializing in the respective oncological fields. Following the issuance of these formal medical records, a secondary team of clinical experts at the department performed a comprehensive manual data mining process. This involved the systematic extraction of key clinical and pathological variables into a structured database format. No formal sample size calculation was performed. Instead, all eligible pathology reports available within the OnkoBank database during the study period were included to maximize the precision of performance estimates and reflect real-world institutional practice.

### Comparative Evaluation Framework and Stochastic Sensitivity Testing

To validate the clinical utility of CIDER, we performed a head-to-head comparison between the automated LLM extractions and the curated database. The evaluation focused on 7 key clinical parameters: sex, T stage, N stage, primary tumor organ, histology, year of surgery, and tumor size.

Before statistical comparison, both datasets underwent terminology normalization using predefined mapping rules established by a clinical expert independent of the manual data extraction process. Stages (T and N), sex, histology, and tumor organs were mapped to a unified English nomenclature. Organ-level mappings were generally direct, whereas histological entities required additional harmonization because reporting terminology frequently differed in specificity and granularity. The normalization process was intentionally conservative; subtype-level diagnoses were not collapsed into broader categories during evaluation. Consequently, more specific model outputs (eg, pancreatic ductal adenocarcinoma) were considered discordant from broader manually curated labels (eg, adenocarcinoma), even when a hierarchical relationship existed between the 2 terms.

The primary metric for performance was extraction accuracy, defined as the percentage of identical values between the manually established database and the CIDER output, excluding cases where manual data were unavailable. In addition to concordance estimates, weighted *F*_1_-scores together with Cohen κ statistics were calculated. For tumor size, tolerance-based agreement thresholds were evaluated.

To assess the impact of model stochasticity on extraction fidelity, the analysis was performed across a range of temperature settings (temperature=0, 0.1, 0.2, 0.5, 1.0, and 2.0). Furthermore, to evaluate the technical reproducibility of the system, the extraction was repeated 3 independent times at the baseline temperature of 0.1.

### Ethical Considerations

Ethics approval was obtained from the Regional and Institutional Research Ethics Committee of Semmelweis University, Budapest, Hungary (88/2021 [2021] and 88-1/2021 [2023]) and the National Centre for Public Health and Pharmacy, Hungary (NNGYK/80409-2/2025).

Written informed consent was obtained from all individual participants included in the study. All patient data were processed locally within an institutionally controlled, secure environment in strict compliance with GDPR guidelines to ensure privacy and confidentiality. No compensation was provided to participants.

## Results

### Descriptive Characteristics of the Validation Cohort

**Figure 2:**
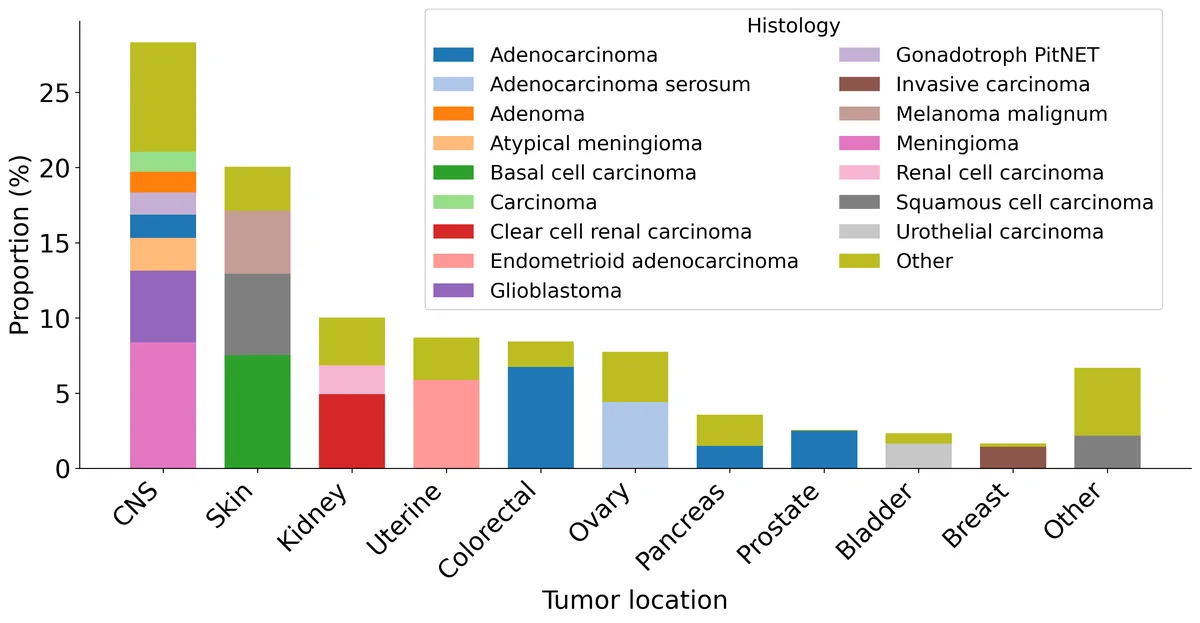
Distribution of tumor locations and histology in the validation cohort. CNS: central nervous system; PitNET: pituitary neuroendocrine tumor.

The validation cohort comprised 2073 histopathology records, with a slightly female-predominant distribution. The temporal distribution of surgical procedures primarily spanned the years 2023 to 2024, accounting for 72.2% (1498/2073) of the total dataset. The database reflects a high-complexity surgical population; among records with available staging, 42.4% (397/937) presented with locally advanced disease (T3-T4), and 32.7% (159/485) demonstrated lymph node involvement (N1-N3). Notably, the manual “gold standard” database contained significant gaps in clinical staging, with T and N stages not reported in 54.8% (1136/2073) and 76.6% (1588/2073) of records, respectively (Table 1; Figure 2).

**Table 1:** Baseline characteristics of the OnkoBank validation cohort (N=2073).

Feature and category			Records, n (%)
**Sex**			
	Not reported	64 (3.1)	
	Female	1112 (53.6)	
	Male	897 (43.3)	
**Year of surgery**			
	Not reported	6 (0.3)	
	2022	203 (9.8)	
	2023	778 (37.5)	
	2024	720 (34.7)	
	2025	366 (17.7)	
**T stage**			
	Not reported	1136 (54.8)	
	T0	28 (1.4)	
	T1	312 (15)	
	T2	200 (9.6)	
	T3	335 (16.2)	
	T4	62 (3)	
**N stage**			
	Not reported	1588 (76.6)	
	N0	326 (15.7)	
	N1	120 (5.8)	
	N2	38 (1.8)	
	N3	1 (<0.1)	
**Tumor size^a^**			
	Not reported	316 (15.2)	

^a^Mean was 36.7 (SD 27.2) mm.

The processed pathology reports consisted of stand-alone PDF documents. Each report corresponded to a single tumor entity of clinical interest, and no multi-document concatenation or patient-level grouping was performed during validation. All evaluated PDFs contained native text, and no optical character recognition pipeline was required during processing. Document structure and formatting varied between contributing institutions and clinical departments. Input document length showed a median of 3926 (mean 4258, SD 1057) tokens, while generated outputs contained a median of 63 (mean 62.5, SD 8.2) tokens. The mean processing time was 14.0 (SD 3.2) seconds per report (median 13.8 seconds), resulting in a total runtime of 2948 seconds for the complete validation cohort. During validation, no invalid JSON outputs, formatting errors, or processing failures were observed.

### CIDER

The CIDER platform [41] provides a streamlined, web-based interface for high-throughput clinical data extraction. The system supports batch processing of up to 1000 PDF documents in a single session. The results are exported as a standardized Excel spreadsheet. Users can interact with the system through an intuitive dashboard that allows for the selection of predefined variables. Users can also define and manage custom extraction variables, for which they must specify a data type (text, numeric, or binary), and a description of the extraction rule, with optional additional parameters (eg, regex pattern constraints for text output).

The validation workflow reported in this study used text-only inputs extracted from native-text PDF files, and Qwen3-VL-32B-Instruct was selected because the broader CIDER platform was designed to support both text-based and multimodal document processing workflows.

The user interface enables granular control over the extraction process through adjustable model parameters, including temperature, top-k, and nucleus sampling (top-p). An additional feature of the CIDER architecture is the inclusion of an extra system prompt, which provides an additional layer of flexibility and allows researchers to provide high-priority instructions. The system also includes a document grouping feature that can automatically concatenate files based on patient-specific prefixes, thereby allowing multi-document records to be analyzed as a single cohesive unit.

The framework supports concurrent processing of multiple uploaded documents and scalable batch processing of large document collections, enabling the analysis of thousands of documents within a single workflow using up to 10 parallel processing threads per session. Structured outputs generation can be constrained through grammar-constrained decoding. To increase robustness, generated JSON outputs undergo automatic parsing and validation; if invalid formatting is detected, regeneration is attempted up to 3 times before the document is skipped. The platform additionally supports multimodal document processing workflows through a vision-language pipeline for image-based or scanned clinical documents.

A critical component of the CIDER is its ability to process multilingual clinical documents. Although the validation cohort consisted of Hungarian-language pathology records, the underlying LLM supports cross-lingual extraction and generation of standardized English outputs. The system also incorporates missing data management logic, providing the option to return “NA” values in the table for missing variables.

### Technical Reliability and Sensitivity to Model Stochasticity

The stability of the CIDER extraction pipeline was evaluated across a range of model temperatures (temperature=0 to temperature=2.0) and through repeated independent runs at temperature=0.1 (Table 2). At the baseline temperature of 0.1, the system demonstrated high reproducibility; categorical variables such as sex (99.50% accuracy) and N stage (92.37% accuracy) showed zero variance across 3 independent repetitions. Minor fluctuations were observed in more linguistically complex fields, such as histology (mean 87.51%, SD 0.05%) and T stage (mean 95.55%, SD 0.06%), though the SD remained negligible, confirming the system’s suitability for high-throughput clinical data extraction workflows.

**Table 2:** Automated extraction accuracy across model temperatures. The table displays the concordance (%) between the manual gold standard and CIDER extractions for 7 clinical variables across a gradient of temperature settings (temperature=0 to temperature=2.0).

Temperature	Organ (n=1886)	Histology (n=2004)	Sex (n=2009)	Year (n=2067)	T stage (n=937)	N stage (n=485)	Size (n=1757)
0, % (n)	95.8 (1806)	87.6 (1753)	99.5 (1999)	98.1 (2027)	95.5 (895)	92.4 (448)	78 (1370)
0.1 (average of 3), mean %	95.8	87.5	99.5	98.1	95.6	92.4	78.1
0.2, % (n)	96.1 (1813)	88.3 (1769)	99.4 (1998)	98.1 (2027)	95.5 (895)	91.3 (443)	79.5 (1397)
0.5, % (n)	95.7 (1804)	88.1 (1765)	99.5 (1998)	98 (2025)	95.5 (895)	92.2 (447)	78.3 (1375)
1.0, % (n)	95.7 (1804)	88.3 (1769)	99.4 (1998)	97.9 (2024)	95.6 (896)	92.4 (448)	78.9 (1387)
2.0, % (n)	95.2 (1795)	87.9 (1761)	99.4 (1998)	97.6 (2018)	95.2 (892)	92.2 (447)	77.4 (1359)

The model exhibited high robustness even at extreme temperatures across the evaluated temperature range, suggesting that extraction was driven primarily by clinically grounded textual evidence and schema constraints rather than stochastic generation effects.

**Figure 3:**
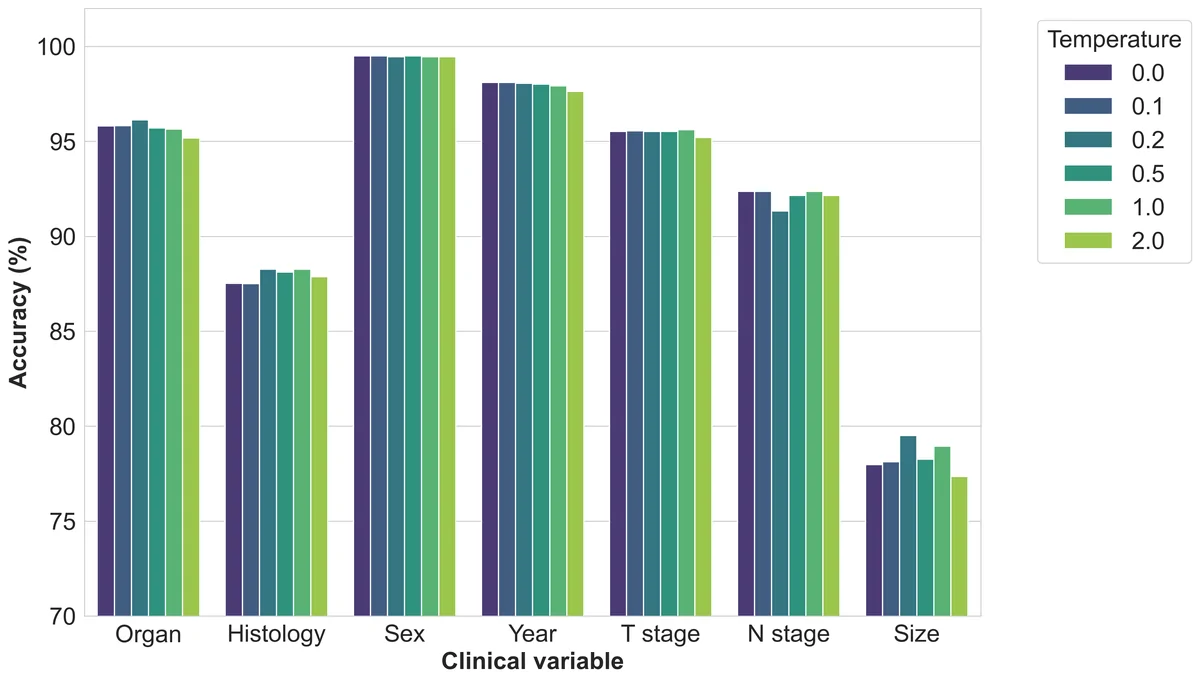
Impact of model temperature on clinical data extraction accuracy. Clustered bar chart illustrates the concordance between the manual gold standard and CIDER (Clinical Data Extractor) outputs across 7 clinical variables. Performance is evaluated across a temperature gradient from temperature=0 (deterministic) to temperature=2.0 (high stochasticity). Accuracy starts at 70% to provide a more granular view of the results.

Notably, performance for histology and size peaked at temperature=0.2 (1769/2004, 88.27% and 1397/1757, 79.51%, respectively), suggesting that a marginal increase in sampling diversity may slightly improve the parsing of highly complex, narrative morphological descriptions. Given that temperature between the temperature=0 and temperature=0.2 interval provided a consistent result, these settings may be suitable for routine high-throughput institutional deployment (Table 2; Figure 3).

The most challenging variable was the maximum tumor diameter (tumor size in mm), which achieved a similar match rate of 77.4% (1359/1757) to 79.5% (1397/1757). Discrepancies in this category were primarily attributed to the high linguistic complexity of histological descriptions, where the computational and manual processing occasionally prioritized different measurements (eg, invasive component size vs total lesion size) when multiple diameters were listed in a single report (Table 2; Figure 3).

Additional agreement analysis demonstrated consistently high weighted *F*_1_-scores and Cohen κ statistics across variables. At the default deployment setting (temperature=0.1), weighted *F*_1_-scores ranged from 0.87 for histology to 0.995 for sex, while Cohen κ values ranged from 0.85 for N stage to 0.99 for sex, indicating substantial-to-near-perfect agreement with the expert-curated reference database. For tumor size extraction, 83.5% (1467/1757) to 85.3% (1498/1757) and 87.3% (1533/1757) to 88.7% (1558/1757) of predictions fell within ±5 mm and ±10 mm of the manually curated values, respectively. Detailed extended evaluation metrics are presented in Multimedia Appendix 3.

### Enhanced Dataset Completeness Through Automated Extraction

Beyond achieving high concordance with existing records, CIDER demonstrated a substantial capacity to recover clinically relevant data points that were omitted during the manual curation process. The manual database encompassed substantial gaps, particularly in pathological staging, where T and N stages were missing in 54.8% (1136/2073) and 76.6% (1588/2073) of cases, respectively.

**Figure 4:**
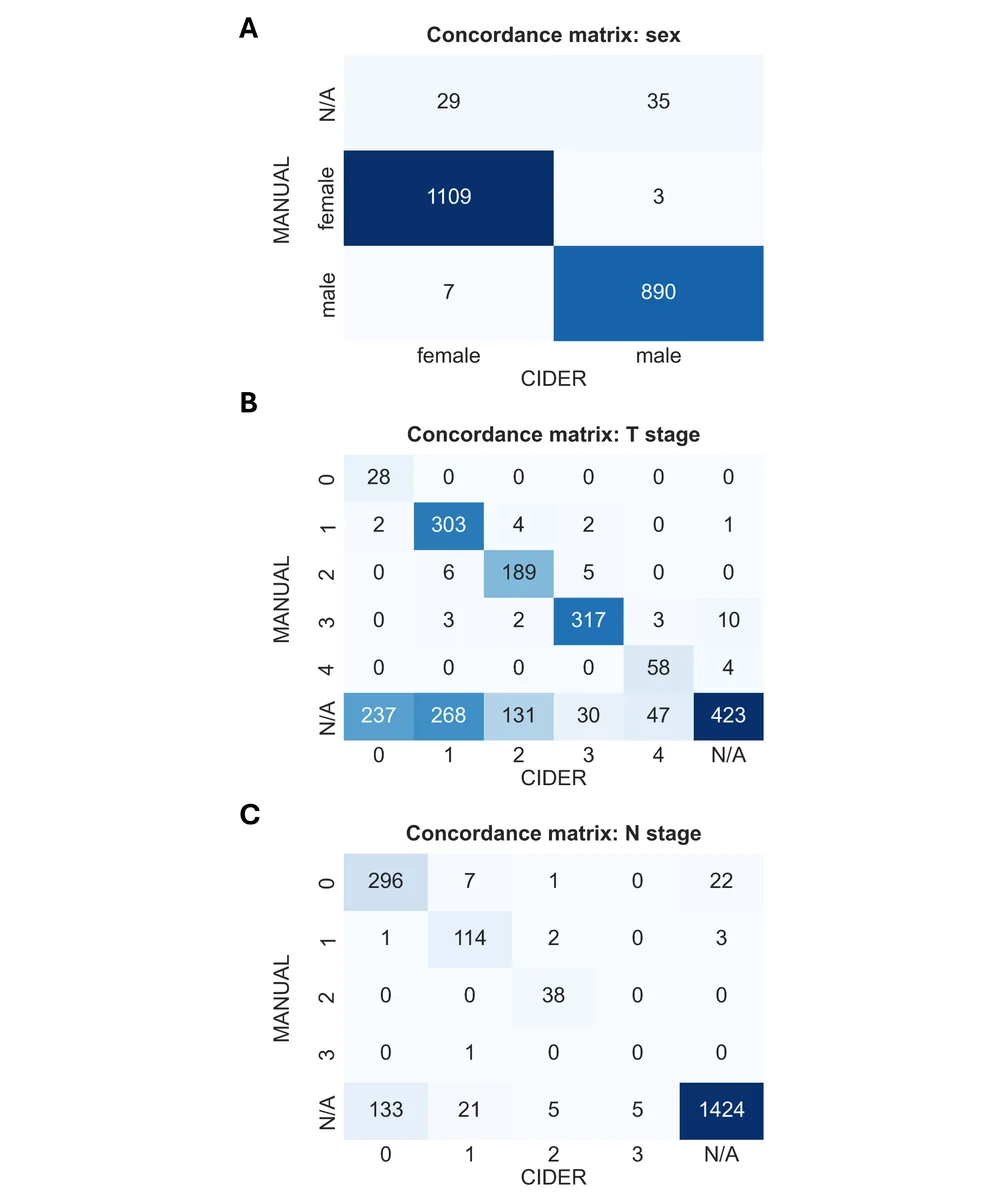
Concordance matrices highlighting the recovered data. Heat maps illustrate the agreement between manual gold standard curation and automated extraction by CIDER (Clinical Data Extractor) for (A) sex, (B) T stage, and (C) N stage. “N/A” values represent missing or nonreported data. The high density along the diagonal confirms exact-match reliability, while the “N/A” row or column intersection reveals the system’s “recovery” capacity and its “refusal” accuracy, where both human and AI correctly identified the absence of reportable information.

CIDER was able to deliver valid values for 62.8% (713/1136) of missing T stages and 10.3% (164/1588) of missing N stages (Table 3; Figure 4). Remarkably, the system also closed data gaps for tumor size and primary organ location, retrieving 289 and 182 additional data points, respectively (Table 3). These findings suggest that automated extraction may help identify clinically relevant information that remains uncaptured during manual curation workflows, particularly in variables with high rates of missingness. However, these candidate extractions were not included in accuracy calculations and were not independently validated against an external reference standard. Therefore, they should be interpreted as candidate annotations rather than verified extraction results, and independent confirmation would be required to before their use in clinical or research applications. Additional confusion matrices for organ classification, histology, year of surgery, and tumor size are presented in Multimedia Appendix 4.

**Table 3:** Gap analysis and data recovery metrics: comparison of data completeness between manual curation and automated extraction using Clinical Data Extractor.

Variable	Manual missing, n	AI recovered, n	Recovery (%)
Organ	187	182	97.3
Histology	69	42	60.9
Sex	64	64	100
Year	6	6	100
T stage	1136	713	62.8
N stage	1588	164	10.3
Size	316	289	91.5

## Discussion

### Principal Results

In this retrospective validation study, we developed and evaluated CIDER as a comprehensive data extraction pipeline that can be integrated into institutional workflows. The system bridges the gap between unstructured clinical documentation and research-ready datasets by allowing investigators to define extraction targets dynamically without requiring task-specific model retraining or manual feature engineering. This design substantially lowers the technical barrier for large-scale clinical data reuse and supports rapid hypothesis generation across diverse oncological research questions [42]. CIDER achieved high agreement with an expert-curated reference database across clinically relevant variables, particularly for sex, surgery year, primary tumor organ, and pathological T and N stages. The system also demonstrated high technical reproducibility across repeated runs and robustness across a broad range of temperature settings.

### Interpretation and Comparison With Literature

Ethical issues related to the deployment of LLMs in health care remain a central concern, particularly with respect to data privacy, governance, and regulatory compliance [27,29,43,44]. Although contemporary “frontier” models such as GPT-5 (OpenAI), Claude (Anthropic), Gemini (Google DeepMind), and Grok (xAI) define the upper bound of performance across general reasoning and instruction-following tasks, their closed-source nature and reliance on third-party, cloud-based deployment necessitate the transfer of sensitive protected health information to external infrastructure [45]. Such practices raise substantial ethical and legal risks and challenges under strict regulatory frameworks, such as the HIPAA (Health Insurance Portability and Accountability Act) and GDPR, effectively prohibiting their use in privacy-sensitive clinical environments [46]. In parallel, a rapidly expanding ecosystem of open-weight LLMs—including DeepSeek, Gemma, GPT-OSS, Mistral, and the Qwen family—has enabled transparent, institutionally deployable alternatives that preserve full data sovereignty [45,47-49]. Within this landscape, and particularly in the 20 to 40 billion parameter class, Qwen3-VL-32B established a strong performance benchmark [33-35], achieving state-of-the-art or near–state-of-the-art results across multimodal reasoning, instruction following, and multilingual understanding. The objective of this study was to validate an institutionally deployable LLM-based extraction pipeline rather than to perform comparative benchmarking across language models. The model’s strong multilingual performance on benchmarks such as Massive Multitask Language Understanding [50], XStoryCloze [51], and MMBench [52] further supported its suitability for applications involving morphologically complex languages. Although formal comparisons against alternative architectures were beyond the scope of this work, future benchmarking studies will be important for determining how model selection influences extraction performance across languages and clinical domains. Furthermore, our pipeline was explicitly designed to operate within an on-premises, air-gapped institutional environment, ensuring regulatory compliance, the elimination of third-party data transfer, and full data security. This deployment strategy primarily affects governance and privacy considerations rather than the underlying extraction performance itself because the inference process remains identical irrespective of whether computation occurs locally or on externally hosted infrastructure.

Rule-based and traditional clinical NLP systems remain important comparators because of their interpretability and established use in well-defined entity extraction tasks [11-14]. However, registry curation often requires more than detecting candidate entities. The same report may contain multiple dates, specimen dimensions, tumor measurements, historical diagnoses, molecular addenda, and anatomical references, making it challenging to identify which information corresponds to the registry variable of interest. In this setting, the main challenge is contextual selection and higher-order interpretation rather than simple pattern recognition [10]. This represents a potential advantage of schema-guided LLM-based extraction, which can use the broader document context to identify the clinically relevant value without task-specific retraining [53]. Nevertheless, standardized benchmarking datasets and direct comparisons with rule-based, traditional machine learning, and LLM-based approaches will be necessary to quantify the incremental value of each methodology [54].

CIDER achieved the highest accuracy when used for well-defined categorical and temporal variables, such as sex and year of surgery, exceeding 98% concordance with the manual reference data. These results are consistent with prior observations that structured or semistructured elements embedded within narrative text are particularly amenable to automated extraction [16,20]. The high agreement observed for sex extraction may be partly explained by the fact that Hungarian given names are typically sex-specific, allowing the model to infer the database-level sex variable from patient names when sex was not explicitly stated in the report. This finding illustrates the ability of LLM-based extraction to use contextual information for variables that are not always presented in an explicitly structured format. Importantly, the system also demonstrated strong performance in more complex oncological variables, including T and N stages and primary tumor organ, which require contextual interpretation rather than simple keyword matching. Accuracy values above 90% for these parameters indicate that modern LLMs can reliably capture clinically meaningful relationships. Notably, many discrepancies in histology were not model failures, as CIDER often provided a more granular diagnosis (eg, specifying pancreatic ductal adenocarcinoma where the manual database only recorded adenocarcinoma), which was flagged as a mismatch despite the model providing a more specific diagnostic description.

The extraction of tumor size proved to be the most challenging task, with an identical match rate of 78.1%. This finding aligns with known difficulties in both manual and automated abstraction of quantitative measurements from pathology reports, where multiple diameters may be reported for different components of a lesion (eg, total tumor size vs invasive component) [55,56]. This underscores a broader methodological challenge in clinical NLP evaluation: apparent disagreement may reflect ambiguity in the source text rather than model failure.

The extraction performance observed in the present study is comparable to that reported in recent pathology and clinical information extraction studies using contemporary LLMs. Recent investigations have demonstrated high accuracy for extraction of pathological staging variables, histological diagnoses, and structured cancer registry elements from free-text pathology reports and other clinical documents [57,58]. Similarly, locally deployed open-weight LLMs have been shown to achieve performance comparable to that of proprietary frontier models while allowing institutions to retain control over sensitive clinical data [59-61]. Importantly, unlike most prior studies that focused primarily on English-language clinical documentation, the present validation was performed using Hungarian pathology reports, demonstrating that high extraction accuracy can be achieved in a morphologically complex and comparatively lower-resource language setting.

An important strength of CIDER is its ability to process non-English clinical documents, as demonstrated by its validation using Hungarian-language pathology reports. Most existing clinical NLP systems and benchmark datasets are heavily English-centric, limiting their applicability in any non–English-speaking health care system [23,62]. Validation in Hungarian is particularly relevant because Hungarian represents a morphologically complex and comparatively lower-resource language. Recent multilingual benchmark studies have demonstrated persistent performance differences between high-resource and lower-resource languages in contemporary LLMs [63].

The rapid evolution of LLMs represents both a challenge and an opportunity for clinical NLP research. Consequently, the primary transferable insight of the present study is not tied to a specific model version but rather to the broader feasibility of institutionally deployable, schema-driven extraction pipelines for transforming unstructured clinical narratives into structured research datasets. The present work therefore provides transferable methodological insights regarding (1) secure on-premises deployment of clinical LLM systems, (2) schema-guided extraction strategies, (3) validation of clinical NLP workflows in morphologically complex and lower-resource languages, and (4) reproducible integration of LLM-based extraction into clinical registry development pipelines. While future model generations will likely improve extraction quality, multilingual robustness, and computational efficiency, the core workflow and validation principles presented here are expected to remain applicable across evolving architectures and document types.

### Limitations

This study has some limitations that should be considered when interpreting the results. Although the underlying Qwen3-VL-32B-Instruct model possesses multilingual capabilities, the present validation was restricted to Hungarian-language pathology reports within the OnkoBank registry framework; therefore, broader applicability across independent cohorts, additional institutions, other languages, and document types requires external validation. Second, while the manually curated OnkoBank database served as a high-quality gold standard, manual data extraction itself is not immune to error. Third, the candidate extractions were clinically plausible extractions not present in the manual database, and these could not be verified against an independent gold standard. Fourth, although the schema-guided architecture of CIDER was designed to support adaptation across extraction tasks, further validation across additional medical specialties and clinical workflows will be necessary to fully assess generalizability.

### Conclusions

In summary, CIDER is an end-to-end, institutionally deployable platform designed to enable secure, scalable, and reliable extraction of structured clinical data from unstructured pathology reports using an LLM. Our results demonstrate that contemporary LLM-based approaches can achieve high agreement with an expert-curated reference database across a range of clinically relevant variables, while simultaneously addressing long-standing limitations of manual data extraction. While objective benchmarks for LLM-based clinical information extraction are still evolving, our results suggest that institutionally deployed LLMs can meet the practical performance requirements for real-world clinical research applications. More broadly, schema-guided and privacy-preserving LLM-based extraction frameworks may facilitate the development of high-quality clinical registries and enable scalable secondary use of routinely collected health care data for research purposes.

## Appendix

Multimedia Appendix 1 The CIDER (Clinical Data Extractor) system prompt.

Multimedia Appendix 2 Predefined schema and variable descriptions.

Multimedia Appendix 3 Extended evaluation metrics across temperature settings.

Multimedia Appendix 4 Confusion matrices for organ, histology, year of surgery, and tumor size extraction.

Multimedia Appendix 5 STARD-AI checklist.

## Abbreviations

BERT: Bidirectional Encoder Representations from Transformers
BioBERT: Bidirectional Encoder Representations from Transformers for biomedical text mining
CIDER: Clinical Data Extractor
cTAKES: clinical Text Analysis and Knowledge Extraction System
GDPR: General Data Protection Regulation
GPU: graphics processing unit
HIPAA: Health Insurance Portability and Accountability Act
LLM: large language model
NLP: natural language processing

## Data Availability

The clinical data analyzed in this study contain sensitive patient information and cannot be made publicly available because of institutional and regulatory restrictions. Summary data supporting the findings of this study are included within the manuscript and its supplementary materials. Requests for additional information may be directed to the corresponding author and will be considered on a case-by-case basis, subject to institutional approval and applicable data protection regulations.
