# Supplementary material for: A Secure, Scalable Large Language Model–Based System (CIDER) for High-Throughput Clinical Data Extraction From Medical Reports: Retrospective Validation Study
Source: J Med Internet Res. 2026 Sep 2;28:e95780. doi: 10.2196/95780 (PMC13583534; doi:10.2196/95780)
Supplement: Supplementary file 2 [file jmir_v28i1e95780_app2.pdf]

Supplementary File 2. Predefined schema and variable descriptions

"name": "Tumor organ",
"datatype": "string",
"description": "Organ of the primary tumor (e.g., breast, lung, colon, etc.), In case of cancer of unknown primary origin (e.g., CUP) record as NA."

"name": "Histology",
"datatype": "string",
"description": "Microscopic classification of tumor tissue according to histological type (e.g., adenocarcinoma, squamous cell carcinoma)."

"name": "T",
"datatype": "numeric",
"description": "Primary tumor extent according to the TNM classification (T0–T4). Describes local invasion depth and size. If no malignant tumor is present at all just carcinoma in situ, premalignant lesion record as 0 and if its dignity is benign, record as -1. If the T stage is not available, record as NA."

"name": "N",
"datatype": "numeric",
"description": "Regional lymph node status as defined by the TNM classification (N0–N3). This field captures the reported N stage if explicitly provided (e.g., ‘N1’, ‘pN2’). If lymph node involvement is described without a stated N category, this field remains null."

"name": "Sex",
"datatype": "string",
"description": "Patient's biological sex (e.g., male, female.)"

"name": "Year of surgery",
"datatype": "numeric",
"description": "Calendar year in which the surgical procedure was performed, corresponding to the operation from which the analyzed pathological specimen was obtained."

"name": "Size",
"datatype": "numeric",
"description": "Largest measured macroscopic dimension of the tumor (in millimeters). Prioritize malignant findings over benign findings, even if the benign findings are larger. In case of multiple malignant tumors, the largest is considered."
