# Supplementary material for: A Secure, Scalable Large Language Model–Based System (CIDER) for High-Throughput Clinical Data Extraction From Medical Reports: Retrospective Validation Study
Source: J Med Internet Res. 2026 Sep 2;28:e95780. doi: 10.2196/95780 (PMC13583534; doi:10.2196/95780)
Supplement: Supplementary file 3 [file jmir_v28i1e95780_app4.pdf]

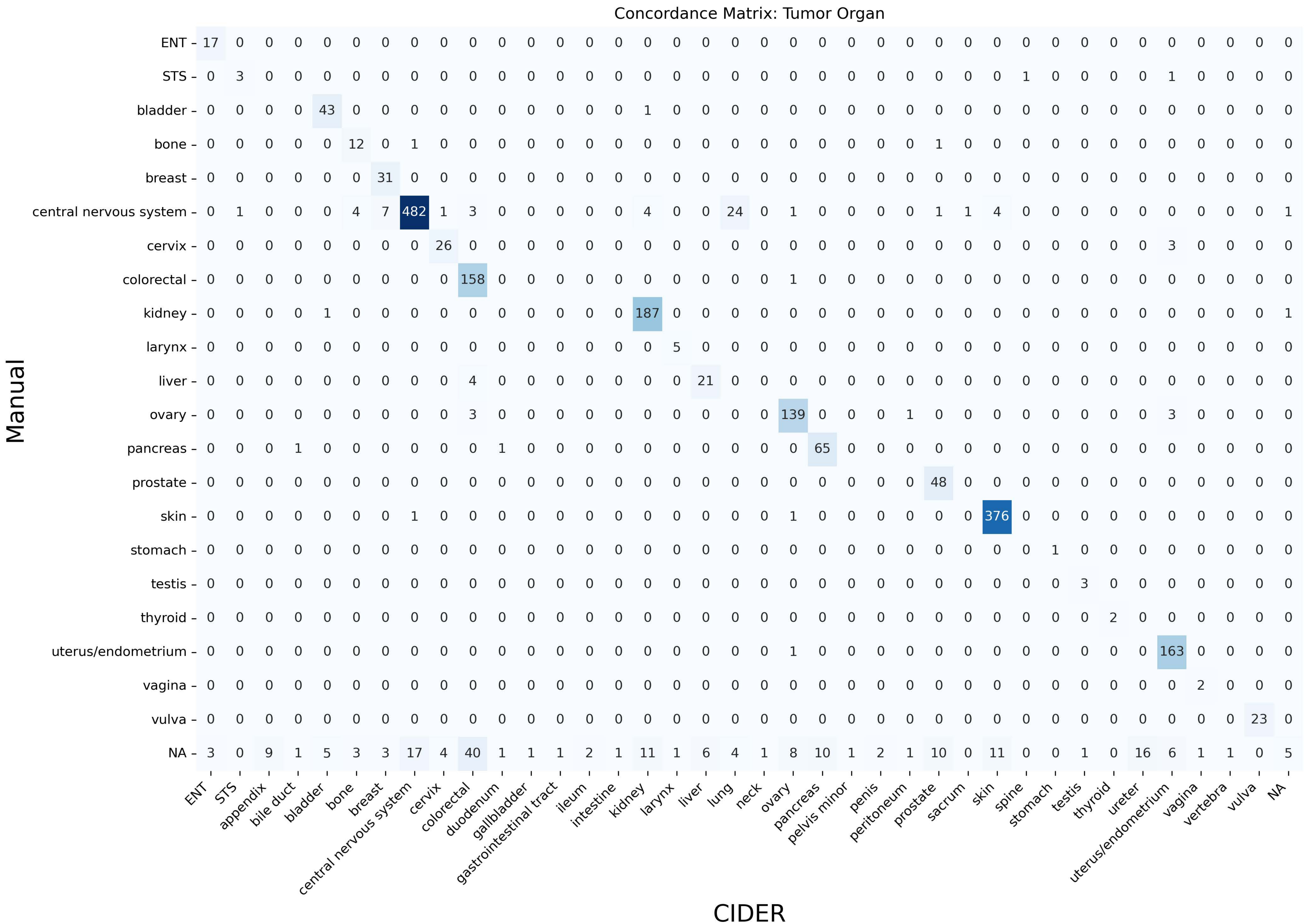
Concordance Matrix: Tumor Organ
Manual
CIDER
ENT
STS
bladder
bone
breast
central nervous system
cervix
colorectal
kidney
larynx
liver
ovary
pancreas
prostate
skin
stomach
testis
thyroid
uterus/endometrium
vagina
vulva
NA
ENT
STS
appendix
bile duct
bladder
bone
breast
central nervous system
cervix
colorectal
duodenum
gallbladder
gastrointestinal tract
ileum
intestine
kidney
larynx
liver
lung
neck
ovary
pancreas
pelvis minor
penis
peritoneum
prostate
sacrum
skin
spine
stomach
testis
thyroid
ureter
uterus/endometrium
vagina
vertebra
vulva
NA

Concordance Matrix: Histology

Rows: Manual; Columns: CIDER

Manual \ CIDER	Other	PDAC	adenocarcinoma	adenocarcinoma mucinosum	adenocarcinoma serosum	adenoma	adenomatosus polyp	astrocytoma	atypical meningioma	basal cell carcinoma	carcinoma	carcinosarcoma	chordoma	clear cell renal cell carcinoma	endometrioid adenocarcinoma	glioblastoma	gonadotroph hypophysis neuroendocrine tumor (PitNET) / adenoma	hepatocellular carcinoma	invasive carcinoma	keratoacanthoma	melanoma malignum	meningioma	neuroendocrine tumor	oligodendroglioma	oncocytoma	papillary renal cell carcinoma	papillary urothelial carcinoma	renal cell carcinoma	schwannoma	squamous cell carcinoma	urothelial carcinoma
Other	313	0	14	2	2	1	2	0	1	4	1	1	0	3	5	2	1	3	1	0	1	3	0	1	0	1	0	0	0	10	0
PDAC	0	11	0	0	0	0	0	0	0	0	0	0	0	0	0	0	0	0	0	0	0	0	0	0	0	0	0	0	0	0	0
adenocarcinoma	4	17	275	4	1	0	0	0	0	0	0	0	0	0	0	0	0	0	0	0	0	0	0	0	0	0	0	0	0	0	1
adenocarcinoma mucinosum	0	0	3	9	0	0	0	0	0	0	0	0	0	0	0	0	0	0	0	0	0	0	0	0	0	0	0	0	0	0	0
adenocarcinoma serosum	0	0	2	0	95	0	0	0	0	0	1	0	0	0	0	0	0	0	0	0	0	0	0	0	0	0	0	0	0	0	0
adenoma	14	0	0	0	0	2	0	0	0	0	0	0	0	0	0	0	6	0	0	0	0	0	5	0	0	0	0	0	0	0	0
adenomatosus polyp	0	0	0	0	0	1	9	0	0	0	0	0	0	0	0	0	0	0	0	0	0	0	0	0	0	0	0	0	0	0	0
astrocytoma	0	0	0	0	0	0	0	17	0	0	0	0	0	0	0	0	0	0	0	0	0	0	0	0	0	0	0	0	0	0	0
atypical meningioma	1	0	0	0	0	0	0	0	27	0	0	0	0	0	0	0	0	0	0	0	0	13	0	0	0	0	0	0	0	0	0
basal cell carcinoma	0	0	0	0	0	0	0	0	0	142	0	0	0	0	0	0	0	0	0	0	0	0	0	0	0	0	1	0	0	1	0
carcinoma	9	0	4	0	16	0	0	0	0	0	18	1	0	3	2	0	0	0	1	0	0	0	0	0	0	0	0	0	0	5	1
carcinosarcoma	0	0	0	0	0	0	0	0	0	0	0	8	0	0	0	0	0	0	0	0	0	0	0	0	0	0	0	0	0	0	0
chordoma	0	0	0	0	0	0	0	0	0	0	0	0	10	0	0	0	0	0	0	0	0	0	0	0	0	0	0	0	0	0	0
clear cell renal cell carcinoma	2	0	0	0	0	0	0	0	0	0	0	0	0	91	0	0	0	0	0	0	0	0	0	0	0	0	0	1	0	0	0
endometrioid adenocarcinoma	0	0	0	0	0	0	0	0	0	0	1	0	0	0	120	0	0	0	0	0	0	0	0	0	0	0	0	0	0	0	0
glioblastoma	1	0	0	0	0	0	0	2	0	0	0	0	0	0	0	86	0	0	0	0	0	0	0	0	0	0	0	0	0	0	0
gonadotroph hypophysis neuroendocrine tumor (PitNET) / adenoma	5	0	0	0	0	8	0	0	0	0	0	0	0	0	0	0	12	0	0	0	0	0	3	0	0	0	0	0	0	0	0
hepatocellular carcinoma	0	0	0	0	0	0	0	0	0	0	0	0	0	0	0	0	0	14	0	0	0	0	0	0	0	0	0	0	0	0	0
invasive carcinoma	3	0	0	0	0	0	0	0	0	0	0	0	0	0	0	0	0	0	24	0	0	0	0	0	0	0	0	0	0	0	0
keratoacanthoma	0	0	0	0	0	0	0	0	0	0	0	0	0	0	0	0	0	0	0	11	0	0	0	0	0	0	0	0	0	0	0
melanoma malignum	0	0	0	0	0	0	0	0	0	0	0	0	0	0	0	0	0	0	0	0	91	0	0	0	0	0	0	0	0	0	0
meningioma	10	0	0	0	0	0	0	0	12	0	0	0	0	0	0	0	0	0	0	0	0	136	0	0	0	0	0	0	0	0	0
neuroendocrine tumor	0	0	0	0	0	0	0	0	0	0	0	0	0	0	0	0	0	0	0	0	0	0	15	0	0	0	0	0	0	0	0
oligodendroglioma	0	0	0	0	0	0	0	0	0	0	0	0	0	0	0	0	0	0	0	0	0	0	0	11	0	0	0	0	0	0	0
oncocytoma	0	0	0	0	0	0	0	0	0	0	0	0	0	0	0	0	0	0	0	0	0	0	0	0	15	0	0	0	0	0	0
papillary renal cell carcinoma	0	0	0	0	0	0	0	0	0	0	0	0	0	0	0	0	0	0	0	0	0	0	0	0	0	16	0	0	0	0	0
papillary urothelial carcinoma	0	0	0	0	0	0	0	0	0	0	0	0	0	0	0	0	0	0	0	0	0	0	0	0	0	0	8	0	0	0	0
renal cell carcinoma	2	0	0	0	0	0	0	0	0	0	0	0	0	13	0	0	0	0	0	0	0	0	0	0	0	1	0	20	0	0	0
schwannoma	0	0	0	0	0	0	0	0	0	0	0	0	0	0	0	0	0	0	0	0	0	0	0	0	0	0	0	0	22	0	0
squamous cell carcinoma	2	0	0	0	0	0	0	0	0	0	0	0	0	0	0	0	0	0	0	0	0	0	0	0	0	0	0	0	0	155	0
urothelial carcinoma	0	0	0	0	0	0	0	0	0	0	0	0	0	0	0	0	0	0	0	0	0	0	0	0	0	0	1	0	0	1	46

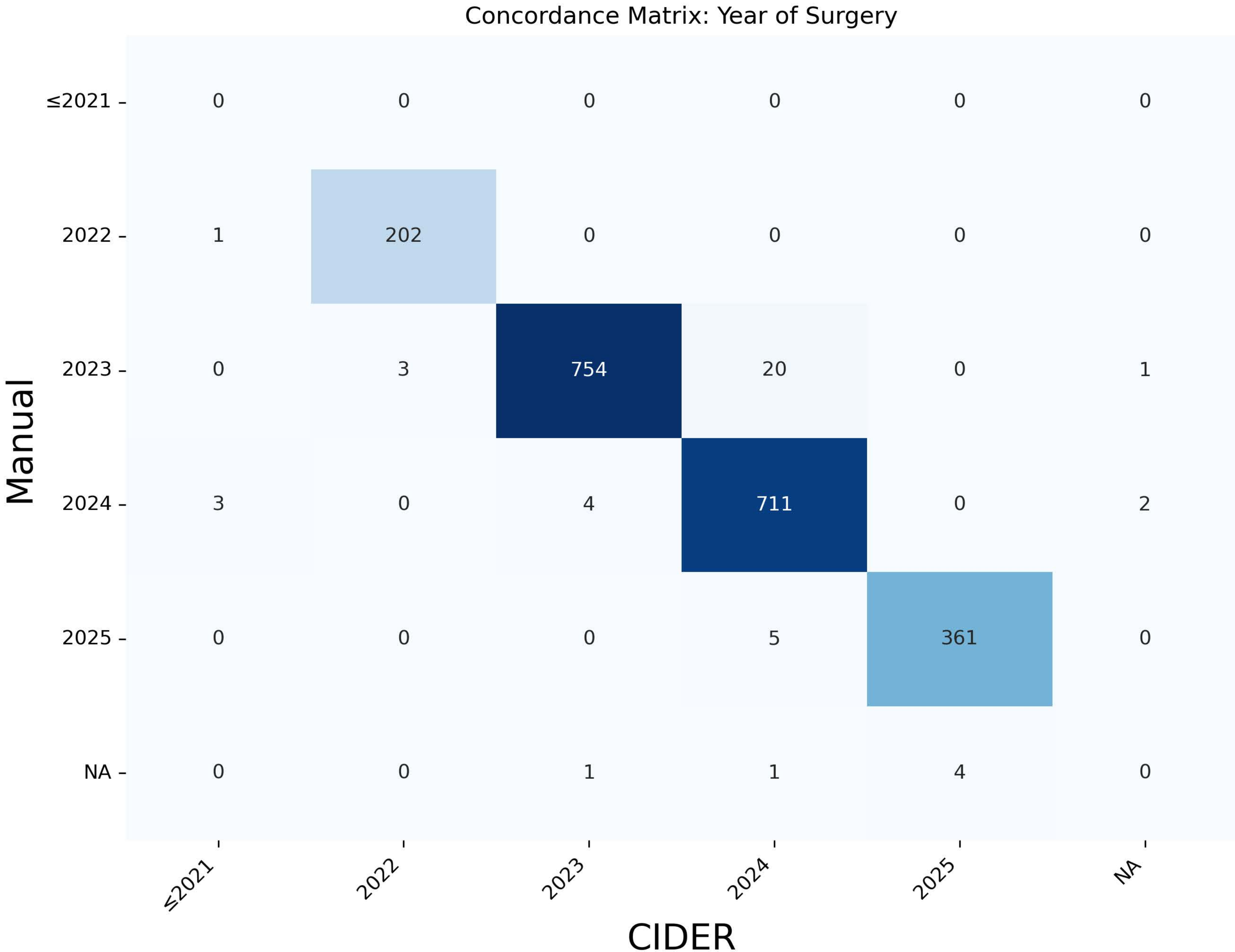
Concordance Matrix: Year of Surgery
Manual
CIDER
≤2021
2022
2023
2024
2025
NA
0 0 0 0 0 0
1 202 0 0 0 0
0 3 754 20 0 1
3 0 4 711 0 2
0 0 0 5 361 0
0 0 1 1 4 0

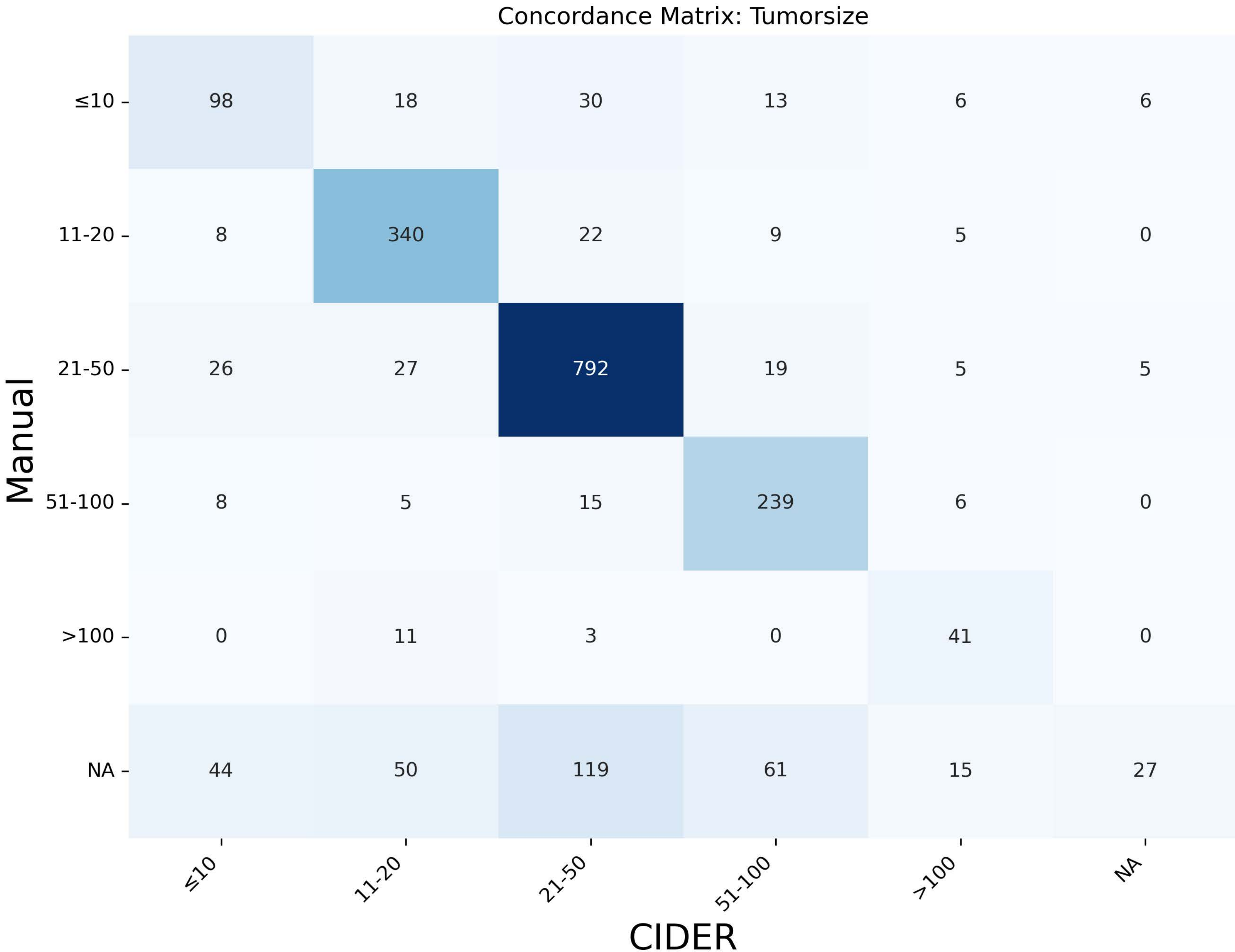
Concordance Matrix: Tumorsize
Manual
CIDER
≤10
11-20
21-50
51-100
>100
NA
98 18 30 13 6 6
8 340 22 9 5 0
26 27 792 19 5 5
8 5 15 239 6 0
0 11 3 0 41 0
44 50 119 61 15 27
