# Supplementary material for: A Secure, Scalable Large Language Model–Based System (CIDER) for High-Throughput Clinical Data Extraction From Medical Reports: Retrospective Validation Study
Source: J Med Internet Res. 2026 Sep 2;28:e95780. doi: 10.2196/95780 (PMC13583534; doi:10.2196/95780)
Supplement: Supplementary file 4 [file jmir_v28i1e95780_app5.docx]

**Supplementary Material 5. STARD-AI Checklist**

| Item | STARD-AI Item | Reported in manuscript |
| --- | --- | --- |
| 1 | Identification as a study reporting AI-centered diagnostic accuracy within title or abstract | Title; Abstract |
| 2 | Structured summary of study design, methods, results, and conclusions | Abstract |
| 3 | Scientific and clinical background, intended use of the index test, and workflow integration | Introduction |
| 4 | Study objectives and hypotheses | Introduction |
| 5 | Retrospective or prospective study design | Materials and Methods – OnkoBank database setup validation |
| 6 | Ethics approval | Ethics approval section |
| 7 | Eligibility criteria | Materials and Methods – OnkoBank database setup validation |
| 8 | Basis for identifying eligible participants | Materials and Methods – OnkoBank database setup validation |
| 9 | Setting, location, and dates of participant identification | Materials and Methods – OnkoBank database setup validation |
| 10 | Whether participants formed a consecutive, random, or convenience series | Materials and Methods – OnkoBank database setup validation |
| 11 | Source of the dataset | Materials and Methods – OnkoBank database setup validation |
| 12 | Reference standard annotations and annotator expertise | Materials and Methods – OnkoBank database setup validation |
| 13 | Hardware and software used for the index test | Materials and Methods – CIDER system architecture |
| 14 | Data acquisition protocols and preprocessing | Materials and Methods – CIDER system architecture |
| 15a | Index test in sufficient detail to allow replication | Materials and Methods – CIDER system architecture; Schema-guided extraction |
| 15b | Development of the index test, including training/validation/testing | Not applicable; externally developed foundation model used without retraining |
| 15c | Definition of test positivity cut-offs or categories | Materials and Methods – Comparative evaluation framework |
| 15d | Intended end users and required expertise | Results – CIDER |
| 16a | Reference standard in sufficient detail to allow replication | Materials and Methods – OnkoBank database setup validation |
| 16b | Rationale for choosing the reference standard | Materials and Methods – OnkoBank database setup validation |
| 16c | Reference standard categories and rationale | Materials and Methods – Comparative evaluation framework |
| 17a | Availability of clinical information/reference standard to index test performers | Not applicable; fully automated extraction pipeline |
| 17b | Availability of index test results to reference standard assessors | Materials and Methods – OnkoBank database setup validation |
| 18 | Statistical methods used to estimate diagnostic accuracy | Materials and Methods – Comparative evaluation framework |
| 19 | Handling of indeterminate results | Results – CIDER; Materials and Methods – Comparative evaluation framework |
| 20 | Handling of missing data | Materials and Methods – Comparative evaluation framework; Results – Enhanced dataset completeness |
| 21 | Analyses of variability in diagnostic accuracy | Materials and Methods – Comparative evaluation framework; Results – Technical reliability and sensitivity analyses |
| 22 | Intended sample size and rationale | Materials and Methods – OnkoBank database setup validation |
| 23 | Error analysis, bias, or fairness assessments | Discussion – Limitations; not formally assessed |
| 24 | Participant flow diagram | The validation cohort consisted of all eligible pathology reports available during the study period, and no additional exclusions were applied. |
| 25 | Baseline demographic, clinical, and technical characteristics | Results – Descriptive characteristics of the validation cohort; Table 1 |
| 26a | Distribution of disease severity | Results – Descriptive characteristics of the validation cohort; Table 1 |
| 26b | Distribution of alternative diagnoses | Not applicable |
| 27 | Time interval and interventions between index test and reference standard | Not applicable |
| 28 | Representativeness of the intended use population | Discussion – Generalizability |
| 29 | External evaluation dataset comparisons | Not applicable; no external validation cohort |
| 30 | Cross-tabulation of index test and reference standard results | Figure 4; Supplementary Material 4 |
| 31 | Estimates of diagnostic accuracy and precision | Results – Technical reliability and sensitivity analyses; Supplementary Material 3 |
| 32 | Adverse events | Not applicable |
| 33 | Study limitations, bias, uncertainty, and generalizability | Discussion – Limitations |
| 34 | Implications for practice and intended clinical role | Discussion – Conclusions |
| 35 | Ethical considerations and fairness | Discussion; Ethics approval |
| 36 | Registration number and name of registry | Not applicable |
| 37 | Access to full study protocol | Not applicable |
| 38 | Sources of funding and role of funders | Funding statement |
| 39 | Commercial interests | Conflict of Interests statement |
| 40a | Availability of datasets and code | Data Availability statement |
| 40b | Whether outputs are stored and auditable | Materials and Methods – CIDER system architecture |
